# Assessing optimal nitrate/ ammonium- ratios in baby-leaf lettuce to enhance the heat stress tolerance under elevated CO_2_ conditions

**DOI:** 10.1371/journal.pone.0278309

**Published:** 2022-11-30

**Authors:** Jacinta Collado-González, María Carmen Piñero, Ginés Otalora, Josefa López-Marín, Francisco M. del Amor

**Affiliations:** Department of Crop Production and Agri-Technology, Murcia Institute of Agri-Food Research and Development (IMIDA), Murcia, Spain; Government College University Lahore, PAKISTAN

## Abstract

In recent years, the interest on baby-leaf lettuce has grown steadily, because it is richer in bioactive compounds than other traditional vegetables. However, the quality of lettuce is being increasingly affected by climate change. It is very rare for a climatic effect to occur in isolation. Even then, a large body of work has only focused on the effect of isolated heat stress, fertilization, and elevated CO_2_, on morphological, physiological and biochemical parameters. Thus, very few works have focused on how the combination of several of these factors can affect these parameters. For first time, the present work studied the combined effect derived from the application of two different levels of CO_2_ (400 and 1000 ppm of CO_2_), four different NO_3_^-^/ NH_4_^+^ ratios (100/0 (T-I), 100/0 before the short-term heat stress and finally without NO_3_^-^ (T-II), 80/20 (T-III) and 50/50 (T-IV)), and a short-term heat stress (25 and 43°C), on some physiological and quality parameters (dry biomass, photosynthetic parameters, pigments content, lipid peroxidation and total soluble proteins content) of baby-leaf lettuce cv Derbi. Additionally, a comparison of that combined effect of all these parameters between inner and outer leaves was also performed. The results obtained indicated that the interaction between the nutrient solution containing a 50/50 NO_3_^-^/ NH_4_^+^ ratio and a high concentration of CO_2_ (1000 ppm) improved the biomass, photosynthesis, intercellular/external CO_2_ concentration ratio (ci/ca), stomatal conductance (gs), evapotranspiration (E) and lipid peroxidation, and protein content in this baby-leaf lettuce. The results obtained in this work lead us to conclude that this existing interaction between the NO_3_^-^/ NH_4_^+^ ratio and the elevated CO_2_ concentration may be considered as a new strategy for making baby-leaf lettuce more resistant to heat stress, in other words, stronger against the ever more frequent heat waves.

## Introduction

Lettuce (*Lactuca sativa* L.) is the most cultivated and consumed green leafy vegetable in the world, mainly due to its outstanding content in health-promoting compounds, such as vitamins, minerals and antioxidant compounds [1]. In recent years, Europeans have started to consume healthier foods [2]. This trend of eating as healthy as possible, and the fact that baby-leaf lettuces are richer in bioactive compounds as compared to other traditional vegetables, has made this type of lettuce increasingly popular [3]. Numerous studies have indicated that the accumulation of these healthy compounds is strongly influenced by the growing conditions, temperature, and nutrient availability in the nutrient solution [4].

Climate change is the main global environmental problem presently faced by humanity. Among all the negative effects from global warming, the increasingly frequent heat waves and drought are considered two of the most damaging [5]. These climatic phenomena magnify the processes of desertification and erosion, and can lead to a generalized loss of biodiversity [6].

An increase of approximately 1°C in global temperature has been observed since the Industrial Revolution. According to experts, this increase is due to the continuous CO_2_ emissions to the environment resulting from the use of fossil fuels such as coal, oil and natural gas, and an increase of 1.8°C to 4.0°C over the current average global temperature is expected to occur by the end of this century [7]. Previous studies have reported that heat stress in plants can cause multiple negative effects on plant growth, development and yield. For this reason, in order to survive in these stressful conditions, several physiological and metabolic alterations, such as inhibition of photosynthesis, protein denaturation, inactivation of enzymes in chloroplasts and mitochondria, and antioxidant activities, occur in plants. Such alterations lead to a higher lipid peroxidation and a lower biomass and reduced content of proteins and pigments, as well [8]. The ability of plants to adapt to heat stress depends on their species and variety, as well as on the severity of the stress [9].

An isolated climatic phenomenon rarely occurs in nature, with the interaction between several natural hazards being the most common situation [10]. A direct consequence of global warming is the incessant and, at the same time, progressive increase in temperature and concentration of CO_2_. Therefore, and in order to obtain a more realistic picture of how changing weather conditions will affect plants, these two factors of global change should always be studied together [11]. Over the last two centuries, the concentration of atmospheric CO_2_ has increased tremendously. It has been predicted that global atmospheric CO_2_ will exceed 800 ppm by the end of the 21st century [12, 13]. In contrast to the response of animals and humans to elevated CO_2_ concentrations, plants seem to show positive results. In fact, it has been reported that under elevated CO_2_ concentration conditions, not only did plants’ growth and yield improved, but also, a greater tolerance of plants to a variety of abiotic stresses, including heat stress, was achieved [14]. However, little information still exists on the response of plants to the combination of heat stress and high CO_2_ concentrations.

In recent decades, as a consequence of the great increase in the world’s food production, the use of nitrogenous fertilizers (N) has also increased [15]. Since nitrate (NO_3_^-^) promotes the growth of plants, NO_3_^-^ fertilizers were the most-often used nitrogen fertilizer. Nonetheless, the excessive use of nitrate fertilizers does not result in greater improvements in plant yield and quality, and is causing agricultural and environmental pollution. Ammonium (NH_4_^+^) is the other important source of N absorption in plants [16]. Previous research has also revealed that the contribution of NH_4_^+^ can help alleviate the adverse effects produced by heat stress [17, 18]. Several studies have reported that the supply of N to the plant in both forms (NO_3_^-^ or NH_4_^+^) has different effects. Mixtures of both forms of N in the nutrient solution led to better results in plants as compared to those obtained when the nutrient solution was formulated with only a single N source (NO_3_^-^ or NH_4_^+^) [19]. In this sense, Xu *et al*. [19] indicated that an appropriate ratio of nitrate to ammonium promoted the synthesis of photosynthetic pigments and enhanced the growth and photosynthesis in lettuce. Likewise, it was reported that an adequate ratio of nitrate to ammonium was conducive to protein synthesis and a reduction in the lipid peroxidation, improving the plant tolerance to several abiotic stresses [17, 19]. The most appropriate NO_3_^-^/ NH_4_^+^ ratio to be used in the nutrient solution may depend on the plant species to be grown [17, 18, 20, 21]. Therefore, the physiological and biochemical metabolism of different crops is affected by metabolic processes such as absorption, storage, transportation, and assimilation, which vary depending on the N source [19].

The starting hypothesis of the present study is that the application of an elevated [CO_2_] and an adequate NO_3_^-^/ NH_4_^+^ ratio should modify some parameters of baby-leaf lettuce, improving its tolerance to heat stress. Thus, the objective of the present study is to evaluate the effects of heat stress, elevated [CO_2_] and nutrient solution with different NO_3_^-^/ NH_4_^+^ ratios, both individually and in combination, on lettuce’s morpho-physiological and biochemical parameters: dry weight, photosynthesis, the intercellular/external CO_2_ concentration ratio (ci/ca), stomatal conductance (g_s_), evapotranspiration (E), lipid peroxidation, and protein content.

## Material and methods

### Experimental conditions, treatments and plant material

Baby-leaf lettuce plants cv Derbi were acquired from a commercial seed supplier (El Jimenado S.A., Murcia, Spain), 21 days after their germination. Then, 60 seedlings of similar size were transplanted into 60 5-L black pots with coconut fiber. Each fertilization treatment contained a total of 20 pots. This experiment was performed in a climate-controlled chamber designed by our research team. The climatic conditions of this study were: 60% relative humidity, photosynthetically active radiation (PAR) of 250 μmol m^-2^ s^-1^ 16/8 h day/night at 28/16°C initially, and 43/30°C during the heat stress period. The PAR was offered by a combination of high-pressure sodium lamps (Son-T Agro;Philips) and fluorescent lamps (TL-D Master reflex 830 and 840; Koninklijke Philips Electronics N.V., Amsterdam, the Netherlands).

This experiment was carried out at two [CO_2_]: 400 ppm CO_2_ and 1000 ppm CO_2_. In order to ensure that the plants did not suffer any episode of drought, drainages were assessed and maintained above 35% every day [22]. A dripper system at a rate of 2 L h^–1^ was used to irrigate the plants. The Hoagland’s nutrient solution used for irrigation consisted of: Ca(NO_3_)_2_·4H_2_O (362.0 mg L^−1^), KNO_3_ (404.4 mg L^−1^), K_2_SO_4_ (131.1 mg L^−1^), MgSO_4_ 7H_2_O (123.2 mg L^−1^), H_3_PO_4_ (0.101 mL) and micronutrients [17]. In this study, four fertilizer treatments with different NO_3_^−^/ NH_4_^+^ ratios were used. These were: Treatment I: 100/0 NO_3_^−^/ NH_4_^+^; Treatment II: 100/0 NO_3_^−^/NH_4_^+^ until the moment immediately prior to the application of short-term heat stress. At that time, all the nitrate intake was replaced by sulfates; Treatment III: 80/20 NO_3_^−^/NH_4_^+^ ratio and Treatment IV: 80/20 NO_3_^−^/NH_4_^+^ ratio. NH_4_^+^ was introduced as (NH_4_)_2_SO_4_ (105.6 mg L^-1^ and 264 mg L^-1^ for the 80/20 ratio treatment, and the 50/50 ratio treatment, respectively).

After thirty-nine days, half of the treated plants in this climate-controlled chamber were completely randomly selected and harvested. All the plants that remained in our climate-controlled chamber were exposed to 43/30°C day / night for 3 days. After that time, all those plants were also collected.

### Chemicals and reagents

Thiobarbituric acid (TBA), Trichloroacetic acid (TCA) and 3,5-Di-tert-4-butylhydroxytoluene (BHT) acquired from Sigma-Aldrich (Steinheim, Germany). These reagents were used for lipid peroxidation determination. Especially, BHT was used as an antioxidant agent in this procedure.

Absolute ethanol, acetone and n-hexane were obtained from Panreac Química (Barcelona, Spain), Scharlau (Barcelona, Spain) and Macron (Center Valley, PA, USA), respectively. Ultrapure water was produced using a Millipore water purification system. Ethanol was used for dissolve the antioxidant agent in the lipid peroxidation protocol, whilst acetone and n-hexane were used in chlorophylls analysis.

### Dry biomass *analysis*

On harvest day, all the plants (five plants per fertilizer treatment) were used to determine biomass. Following a previously-described procedure [23], after weighing the shoot of the intact lettuce, baby-leaf lettuces were divided into inner (internal part) and outer leaves (external part). Part of the plant material was weighed for fresh shoot weight (FW) determination, freeze-dried for 4 days at 70°C, and then weighted again to determine the dry shoot weight (DW).

### Gas exchange measurements

Gas exchange parameters (net CO_2_ assimilation rate (A_CO2_), internal CO_2_ concentration (Ci), stomatal CO_2_ conductance (g_s_), and evapotranspiration (E)) were measured using a CIRAS-2 portable photosynthesis system (PP system, Amesbury, MA, USA) with a PLC6 (U) Automatic Universal Leaf Cuvette.

These parameters were measured following a procedure previously reported by our work team [17], on a fully-expanded mature leaf from each plant. At a leaf temperature of 25°C, the cuvette used provided light (LED) with a photon flux of 1300 μmol m^−2^ s^−1^. These measurements were executed both in the thirty-ninth day and the final day of the total experiment.

### Maximum potential quantum efficiency of photosystem II

Chlorophyll fluorescence parameters were measured according to Del Amor *et al*. [22], using an ADC Fim 1500 system (ADC BioScientific, Herts., England). These measurements were performed after 30 minutes of dark adaptation of one leaf from each plant. The dark conditions were provided by a special leaf clip holder. The measured parameters were as follows: the variable fluorescence from a dark-adapted leaf (Fv), the maximal fluorescence from a dark-adapted, youngest fully-expanded leaf (Fm), and the maximum quantum yield of PSII in dark-adapted leaves (Fv/Fm).

### Measurement of lipid peroxidation

Lipid peroxidation was measured as the amount of thiobarbituric acid-reactive substances (TBARS) determined by the thiobarbituric acid (TBA) reaction [24]. Fresh outer and inner-leaf material (0.1 g) were homogenized with a solution of trichloroacetic acid (TCA) 20% (*w/v*) (3 mL) and centrifuged at 3500 × g for 20 min. An assay mixture containing 1 ml aliquot of the supernatant and 1 ml of 20% (w/v) trichloroacetic acid (TCA) containing 0.5% (w/v) thiobarbituric acid (TBA) and 150μL of 4% BHT (w/v) ethanol was heated to 95°C for 30 min and then rapidly cooled in an ice-bath. Subsequently, the samples were centrifuged at 4°C (10000 x g for 15 min). The supernatant absorbance (532 nm) was read and values corresponding to non-specific absorption (600 nm) were subtracted. The TBARS content was estimated according to its molar extinction coefficient (155 mM^–1^ cm^–1^).

### Photosynthetic pigments

Chlorophylls a and b were extracted from fresh outer and inner-leaf material. Samples (1 g) were homogenized with a solution of acetone–hexane (2:3) (25 mL) by using a Polytron, and centrifuged for 6 min at 5000 x g and at 4°C. The absorbance of the supernatant was measured with a UV/VIS spectrophotometer (Shimadzu UV-1800 model with the CPS-240 cell holder, Shimadzu Europa GmbH, Duisburg, Germany) for chlorophyll a and b at A663, A645, 505 and A453, and concentrations were calculated using equations outlined by Nagata and Yamashita [25]:

$$Chlorophyll\;a\;(mg\;100\;mL^{‐1})=0.999\;*\;A663–0.0989\;*\;A645$$

$$Chlorophyll\;b\;(mg\;100\;mL^{‐1})=‐0.328\;*\;A663+1.77\;*\;A645$$

$$Lycopene\;(mg\;100\;mL^{‐1})=‐0.0458\;*\;A663+0.204\;*\;A645+0.372\;A505–0.0806\;A453.$$

$$Β‐Carotene\;(mg\;100\;mL^{‐1})=0.216\;*\;A663-1.22\;*\;A645–0.304\;A505–0.452\;A453.$$

### Determination of total protein content

Total protein was measured in freeze-dried outer and inner-leaf samples by using a combustion nitrogen/protein analyzer (LECO FP-528, Leco Corporation, St. Joseph, MI, USA) [18].

### Statistical analysis

The data were tested for homogeneity of variance and normality of distribution. The experimental design was completely randomized with a 4 x 2 x 2 x 2 factorial scheme, which was composed by four nitrogen sources (different NO_3_^-^/ NH_4_^+^ ratios: 100/0 (T-I), 100/0 before applying short-term heat stress and finally without NO_3_^-^ (T-II), 80/20 (T-III) and 50/50 (T-IV)), two temperatures (25 and 43°C), two [CO_2_] levels (400 and 1000 ppm CO_2_) for photosynthetic parameters and two different leaf ages (inner and outer leaves). The analyses were performed using five repetitions per treatment. An analysis of variance (ANOVA) was performed, and the means were compared using Tukey’s multiple range test at P ≤ 0.05 with the SPSS software v.21 (IBM, Chicago, IL, USA).

## Results and discussion

### Dry biomass

**Fig 1:**
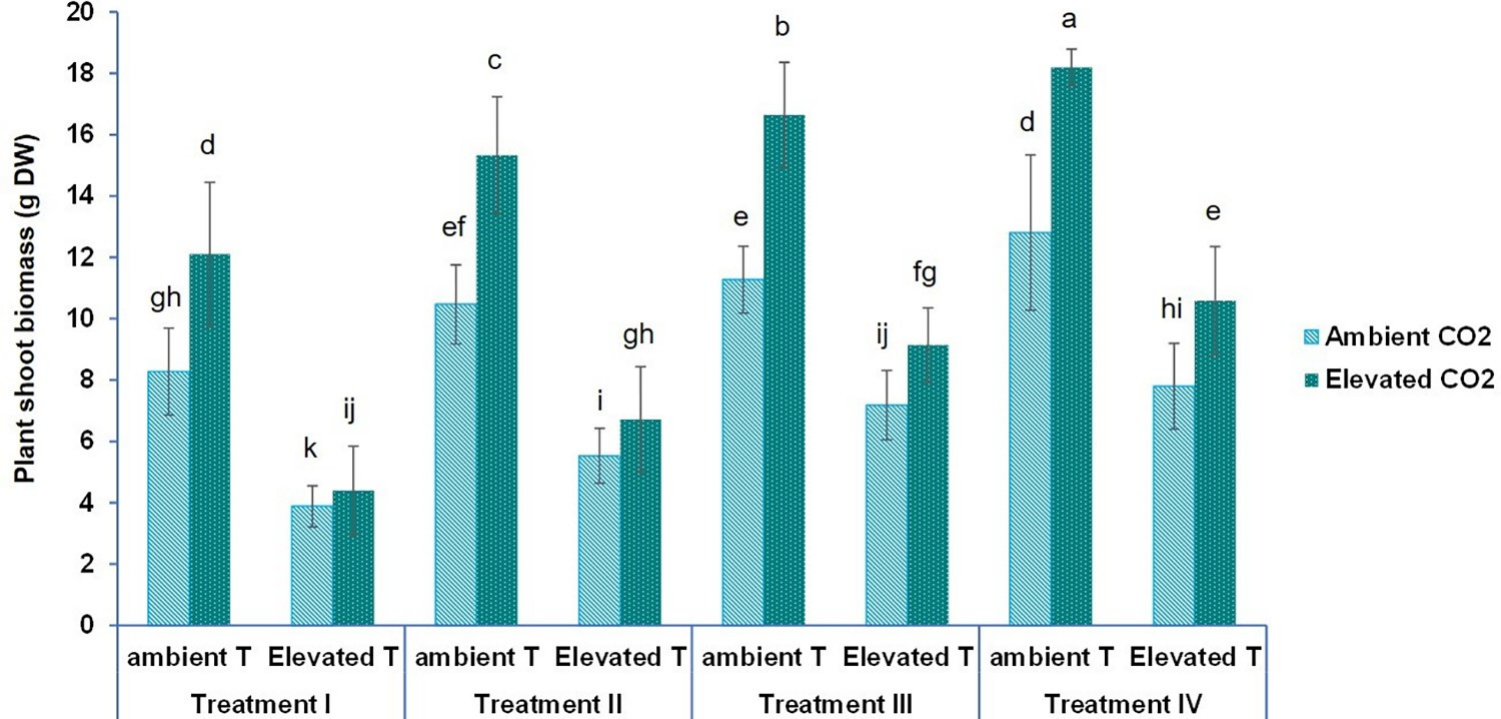
Effect of short-term heat stress, Two levels of [CO_2_] and four different NO3-/NH4+ treatments (T-I: 100/0 NO3− / NH4+; T-II: 100/0 NO3− / NH4+ before short-term heat stress, when all the nitrate intake was replaced by sulphates; T-III: 80/20 NO3− / NH4+ ratio and T-IV: 50/50 NO3− / NH4+ ratios) on total dry weight of baby-leaf lettuce cv Derbi. Different letters are significantly different according to Tukey’s test at p ≤ 0.05.

As shown in Fig 1, noticeable differences were observed between the different treatment groups. In this sense, whilst the lowest shoot dry biomass (3.88 g) was found in plants grown at ambient [CO_2_], fed with the T-I nutrient solution (100/0 NO_3_^−^ / NH_4_^+^) and after short-term heat stress (43°C), the highest value of dry mass (18.19 g) was obtained in plants grown at ambient temperature (28°C), elevated [CO_2_] and with the T-IV nutrient solution. The latter contained the lowest ratio of NO_3_^-^/ NH_4_^+^.

Short-term heat stress led to a decrease of 53.06% in the dry shoot biomass of baby-leaf lettuce cv Derbi in plants under CO_2_ ambient and fed with T-I: (100 NO_3_^-^) (Fig 1). Similar results were observed in a study carried out with Italian lettuce [26]. Paul et al. [27] indicated that an exhaustion of carbohydrate reserves can be caused by heat stress. It has been documented that there is a relationship between the loss of biomass due to heat stress, and a lower capacity for assimilation of compounds [7]. This lower assimilation capacity is mainly due to the fact that heat stress alters the stability of membranes, resulting in a lower rate of photosynthesis and an improvement in the maintenance of respiration costs [28].

In conditions of elevated [CO_2_], dry biomass increased by 46.4% as compared to the ambient temperature when T-I was applied (Fig 1). This result is in accordance with those obtained in many studies carried out in growth chambers, glasshouses, and open-top chambers. These studies confirmed that elevated CO_2_ has an important enhancing effect on biomass and photosynthesis in C3 plants [29].

The plants treated under the four different nitrogen treatments at ambient temperature and ambient [CO_2_] showed an increase in their dry biomass by 26.5, 36.3 and 54.8% as compared to the control (T-I was considered as the control for the nutrient solution). The effect of short-term heat stress was stronger on their dry biomass than the nutrient solution. However, the elevated [CO_2_] boosted this increase in dry biomass caused by different NO_3_^-^/ NH_4_^+^ ratios in the nutrient solution (Fig 1). Many previous studies carried out on sweet pepper (*Capsicum annuum* L) [30], Chinese cabbage (*Brassica campestris* ssp) [31], Canola (*Brassica napus*) [32], pak choi (*Brassicca rapa chinensis*) [33], *Arabidopsis thaliana* [34], and lettuce (*Lactuca sativa*) [19] have shown that the biomass of different crops and plants is promoted by a mixture of NO_3_^-^/ NH_4_^+^ in their nutrient solution. Although the biomass of all those crops was not promoted by the same NO_3_^-^ / NH_4_^+^ ratio in the nutrient solution, the biomass of all of them was optimized by using the 80/ 20 and 50/50 NO_3_^-^ / NH_4_^+^ ratios.

The three-way ANOVA revealed a significant interaction effect between short-term heat stress, [CO_2_], and moderate NO_3_^-^/ NH_4_^+^ ratio in the nutrient solution, on dry biomass (Fig 1 and Table 1).

**Table 1:** ANOVA analysis of how affect the level of CO_2_, short-term heat stress and different NO3-/NH4+ ratios in the nutrient solution on photosynthetic parameters, chlorophylls, lipid peroxidation and proteins and the effect of this factors together with leaves age on chlorophylls, lipid peroxidation and proteins.

Factors	Biomass	A_CO2_	Ci/ Ca	g_s_	E	Fv/Fm	Chl a	Chl b	Chl a+ b	Lipid perox.	Prot
NO_3_^-^/NH_4_^+^ ratio (Tr)	***	***	***	***	***	***	***	***	***	***	***
Temperature (T)	***	***	***	***	***	***	***	***	***	***	***
CO_2_ level (CO_2_)	***	***	***	ns	***	***	***	***	***	***	***
Age	-	-	-	-	-	-	***	***	***	***	***
Tr * T	ns	***	***	***	***		***	*	***	***	***
Tr * CO_2_	***	***	***	***	***	ns	***	ns	***	***	***
Tr * Age	-	-	-	-	-	-	***	ns	***	***	***
T * CO_2_	**	***	**	***	***	ns	***	ns	***	***	***
T * Age	-	-	-	-	-	-	***	*	***	***	Ns
CO_2_ * Age	-	-	-	-	-	-	***	**	***	***	***
Tr * T * CO_2_	**	ns	***	***	***	ns	***	**	**	***	Ns
Tr * T * Age	-	-	-	-	-	-	***	ns	***	***	**
Tr * CO_2_ * Age	-	-	-	-	-	-	***	***	***	***	Ns
T * CO_2_ * Age	-	-	-	-	-	-	***	ns	***	***	**
Tr * T * CO_2_ * Age	-	-	-	-	-	-	***	ns	***	***	*

Values are the means of five replicate samples; means within columns separated using Tukey’s multiple range test. P = 0.05; ns–non significant.

*. ** and ***–significant at P ≤ 0.05. 0.005 and 0.001. respectively.

### Leaf gas exchange

**Fig 2:**
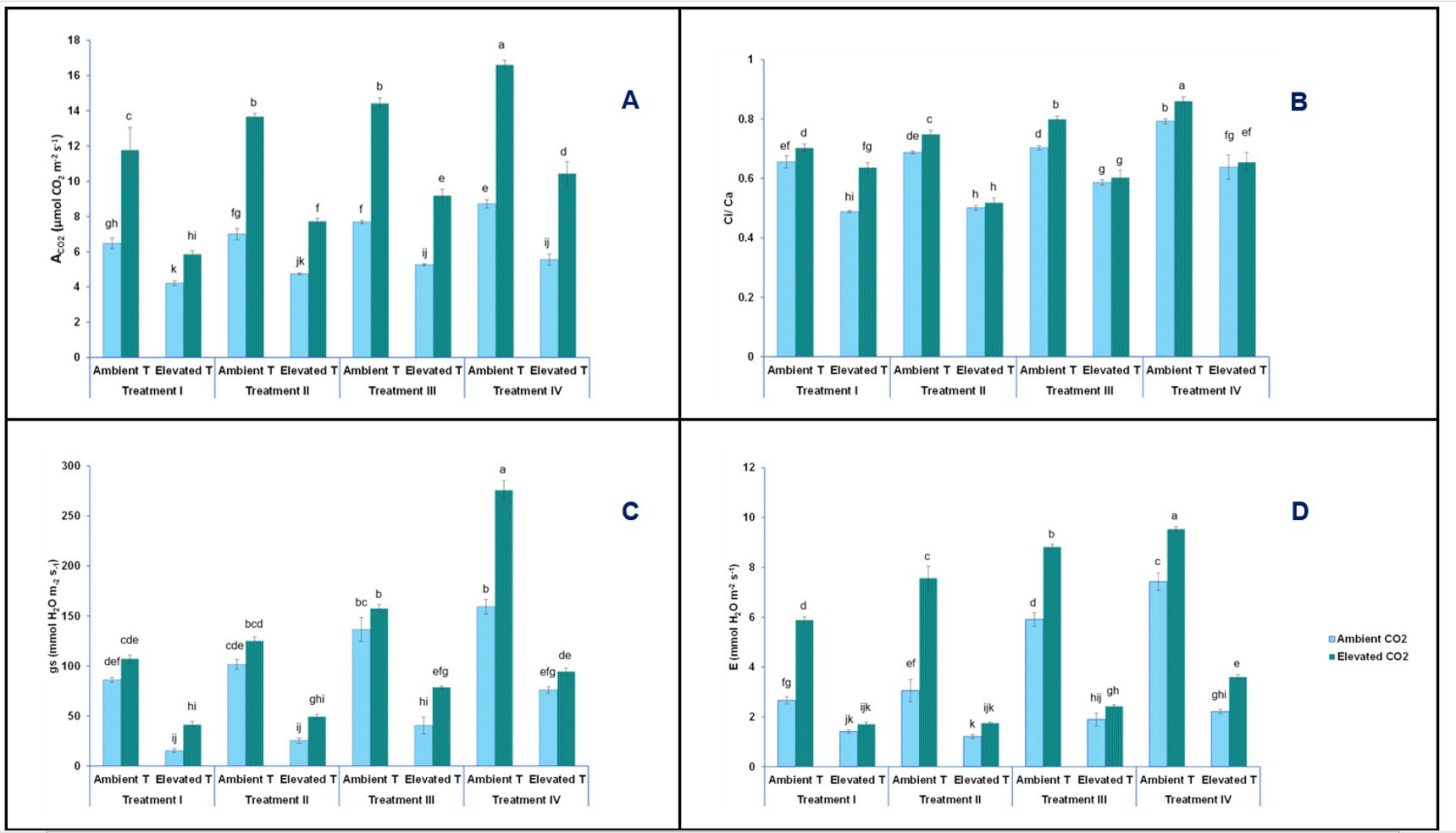
Effect of the short-term heat stress on net photosynthetic rate (A_CO2_) (A), intercellular/external CO_2_ concentration ratio (Ci/Ca) (B), stomatal conductance (g_S_) (C) and evapotranspiration (E) (D) in leaves of baby-leaf lettuce cv Derbi at two levels of CO_2_ and four different NO_3_^-^/NH_4_^+^ treatments. Different letters are significantly different according to Tukey’s test at p ≤ 0.05.

The net CO_2_ assimilation (A_CO2_) followed the same pattern as biomass. In other words, A_CO2_ was affected by short-term heat stress, a high level of CO_2_, and a decreased NO_3_^-^/ NH_4_^+^ ratio. In this sense, A_CO2_ increased from 6.5 μmol m^–2^ s^–1^ at ambient temperature, 400 ppm CO_2_, and T-I nutrient solution (control), to 16.6 μmol m^–2^ s^–1^ at ambient temperature, 1000 ppm CO_2_, and T-IV nutrient solution (Fig 2A). In contrast, the net CO_2_ assimilation rate decreased in plants from 6.5 μmol m^–2^ s^–1^ to 4.2 μmol m^–2^ s^–1^ due to the effect of the short-term heat stress on plants under the same conditions of CO_2_ (ambient CO_2_) and feeding (T-I nutrient solution) (Fig 2A). Following the same trend as photosynthesis, the intercellular/external CO_2_ concentration ratio (ci/ca), stomatal conductance (g_s_) and evapotranspiration (E), increased 1.5, 3 and almost 4-fold, respectively, when plants were grown under ambient temperature and CO_2_ (400 ppm), and when fed with the T-I nutrient solution, as compared with those plants also grown in the absence of short-term heat stress but under elevated levels of CO_2_ and fed with the T-IV nutrient solution. In contrast, reductions of 1.5, almost 6 and 2-fold, respectively, were observed as the action of only the short-term heat stress. Thus, plants were under the CO_2_ (400 ppm) conditions and were fed with the same nitrogen treatment (T-I) (Fig 2B–2D). Thus, as in the biomass results, our data also indicated that an elevated [CO_2_] and temperature, and lower NO_3_^-^/ NH_4_^+^ ratio in the nutrient solution had profound effects on photosynthetic parameters in the studied plants (Table 1).

Our findings from the short-term heat stress were consistent with those previously reported for other vegetables, such as tomato (*Solanum lycopersicum*), soybean (*Glycine max*), wheat (*Triticum aestivum*), maize (*Zea mays*), or rice (*Oryza sativa*) [13, 31]. Studies performed on a large number of vegetables, including lettuce, have attributed a significant decrease in photosynthesis to the lack in the ability of Rubisco activase to maintain Rubisco (the main enzyme responsible for the fixation of CO_2_ in plants) in its active form [8, 35]. A high [CO_2_] can reduce the inhibition of Rubisco carboxylation activity, leading to a higher plant tolerance to heat stress [8, 35, 36]. In addition to observing this dampening effect in our results, this has also been previously observed in other species of plants, including pea (*Pisum sativum* L.), lamb’s quarters (*Chenopodium album* L.), wheat (*Triticum aestivum* L.,), soybean (*Glycine max* L.), sunflower (*Helianthus annuus* L.) and tomato (*Lycopersicon esculentum* L.) [13, 37]. Slattery and Ort [29] indicated that although the elevated [CO_2_] alleviated the negative effects produced by high temperatures, the interaction of both factors (temperature and CO_2_ level) did not reach the values found when only the CO_2_ factor was applied.

Regarding the effect of the NO_3_^-^/ NH_4_^+^ ratio on photosynthetic parameters (Fig 2), our results were in agreement with the findings from other authors, who reported that plant species fed with a higher NH_4_^+^/ NO_3_^-^ ratio showed an improved tolerance to different abiotic stresses [32, 34, 38]. There is a controversy regarding the effects of ammonia on plant photosynthetic parameters. Thus, while some researchers found that ammonia was toxic to plants due to a decrease in photosynthetic parameters, others found the opposite result, and postulated that ammonia was beneficial for some physiological processes performed in chloroplasts [39]. Some studies carried out in some plants, in order to establish a relationship between the N supply and enzymatic activities, reported that ammonium inhibited the nitrate reductase enzyme. Most of those studies hypothesized that as a consequence of such inhibition, the relationship found between leaf photosynthetic rate and nitrogen supply followed a quadratic curve [38]. In this way, until the nitrogen supply or nitrogen content of the leaves reaches a threshold value, photosynthesis grows. But, when this threshold is exceeded, photosynthesis decreases [40]. Considering this hypothesis, together with the results obtained from the biomass, we can deduce that the 50/50 NO_3_^-^/ NH_4_^+^ ratio was not harmful for baby-leaf lettuce cv Derbi, from the physiological point of view.

### Lipid peroxidation

**Fig 3:**
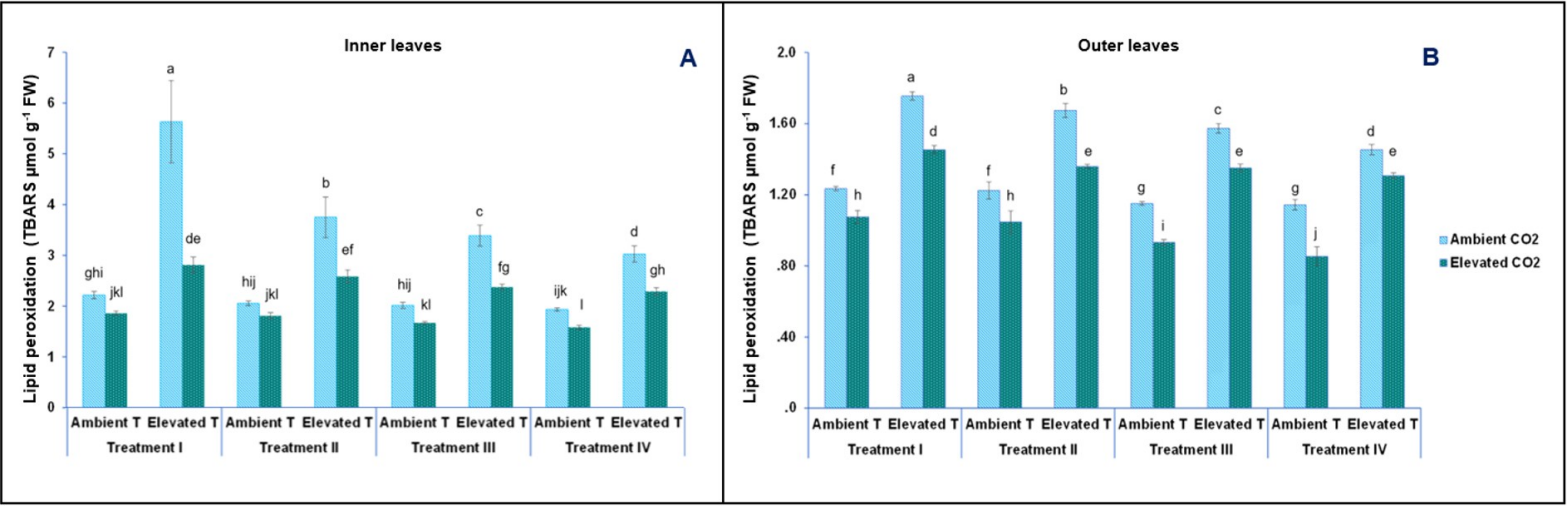
Effect of the short-term heat stress on lipid peroxidation (TBARS μmol g^-1^ FW) in inner (A) and outer (B) leaves of baby-leaf lettuce cv Derbi at two levels of CO_2_ and four different NO_3_^-^/NH_4_^+^ treatments. Different letters are significantly different according to Tukey’s test at p ≤ 0.05.

Lipid peroxidation was measured in inner and outer lettuce leaves under ambient and under elevated [CO_2_], different NO_3_^-^/ NH_4_^+^ ratios in the nutrient solution, and after a short-term heat stress, with the results shown in Fig 3. Lipid peroxidation in the outer lettuce leaves varied significantly from 0.85 TBARS μmol g^-1^ FW, obtained in plants grown under elevated [CO_2_], fed with the T-IV nutrient solution, and at ambient temperature, to 1.76 TBARS μmol g^-1^ FW in leaves from plants grown under ambient [CO_2_], fed with the T-I nutrient solution, and after short-term heat stress. In inner leaves, lipid peroxidation values also increased from 1.58 TBARS μmol g^-1^ FW, in leaves from plants grown under the following conditions: elevated [CO_2_], fed with the T-IV nutrient solution, and ambient temperature, to 5.63 TBARS μmol g^-1^ FW, in leaves from plants grown under ambient [CO_2_], fed with the T-I nutrient solution, and after short-term heat stress (Fig 3).

In plants, we find the paradox that 1% of the oxygen they consume to live is used to produce reactive oxygen species (ROS), such as superoxide O_2_^−^, hydroxyl radical OH^−^, alkoxyl radicals, hydrogen-peroxide H_2_O_2_, and singlet oxygen ^1^O_2_ [41]. These ROS are very reactive and able to damage proteins, lipids, and carbohydrates, generating oxidative stress and its concomitant senescence process [42]. Thus, due to these oxygenation conditions, together with the abundance of photosensitizers and polyunsaturated fatty acids in the chloroplast envelope, photosynthesizing plants are at continuous risk of oxidative damage [40]. Additionally, after heat stress in non-heat tolerant plants, ROS production increases, with higher values of lipid peroxidation observed, indicating that these plants are suffering from a higher oxidative damage [43]. Therefore, the increase in lipid peroxidation resulting from short-term heat stress and the decrease in lipid peroxidation due to a higher [CO_2_], was an expected result (Fig 3 and Table 1). Some studies claim that there is a direct relationship between abiotic stress, including heat and high [CO_2_], tolerance, and increased activities of antioxidant enzymes, particularly superoxide dismutase (SOD), catalase (CAT), and ascorbate peroxidase (APOX), and the prevention of lipid peroxidation of cell membranes [4, 7, 43]. Alternatively, and possibly in a complementary manner, a high [CO_2_] also reduces the amount of ROS by increasing the carboxylation capacity of Rubisco, reducing photorespiration [44]. In addition to reduced photorespiration, a decrease in the activity of NADPH oxidase is also produced. Through the reduction in both activities, a remarkable decrease in H_2_O_2_ levels could be achieved. This may be reflected in reduced lipid peroxidation, and, consequently, a lower degradation of the photosystem [45].

It has been proven that the activities of these anti-oxidant enzymes (SOD, CAT and peroxidases) are highly affected by the N forms present in the nutrient solution, showing a higher activity in plants fed with a NO_3_^-^/NH_4_^+^ ratio than when they are fed with only NO_3_^-^ or NH_4_^+^ [46].

As observed, lipid peroxidation was statistically affected by the age of the leaves (Table 1). Thus, the highest lipid peroxidation was observed in outer leaves, with the lowest measured in inner leaves (Fig 3). This is consistent with other results found, which indicated that old leaves from plants under abiotic stress showed a higher lipid peroxidation and a lower accumulation of antioxidant compounds [47, 48]. The higher accumulation of antioxidant compounds in inner leaves could indicate a premature or accelerated senescence of mature leaves [49].

### Photosystem II maximum efficiency (Fv/Fm) and pigment content

**Fig 4:**
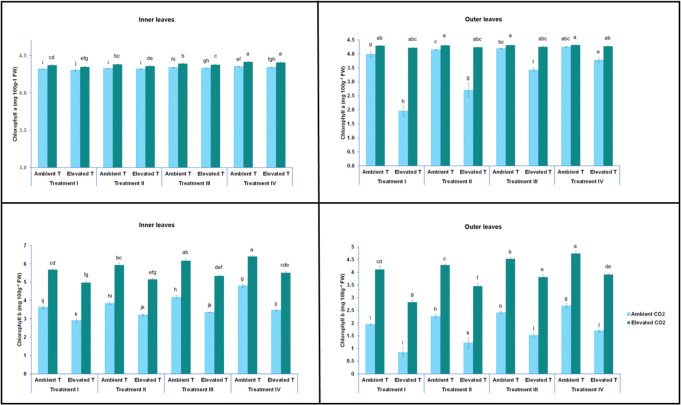
Effect of short-term heat stress on concentrations of chlorophyll a in inner (A) and outer (B) leaves and chlorophyll b (mg L^-1^ cm^-2^) in inner (C) and outer (D) leaves of the baby-leaf lettuce cv Derbi at different levels of CO_2_ and at four different NO_3_^-^/ NH_4_^+^ treatments. Different letters are significantly different according to Tukey’s test at p ≤ 0.05.

**Fig 5:**
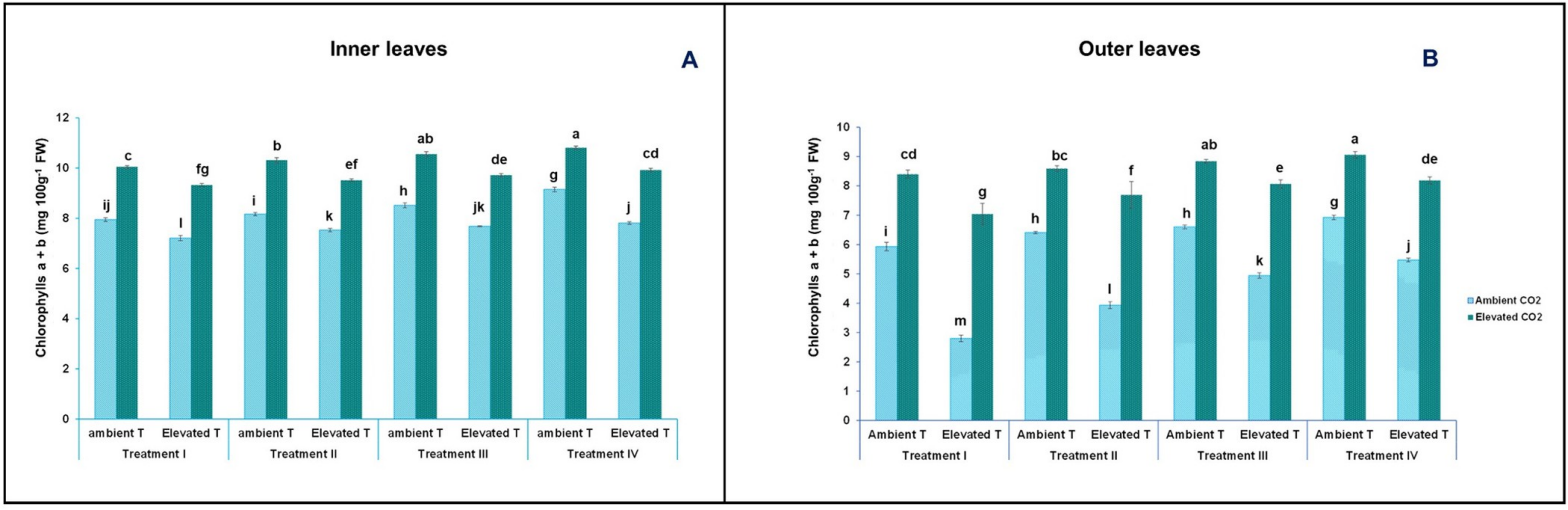
Effect of short-term heat stress on concentrations of chlorophylls a + b in inner (A) and outer (B) leaves of the baby-leaf lettuce cv Derbi at two different levels of CO_2_ and at four different NO_3_^-^/ NH_4_^+^ treatments. Different letters are significantly different according to Tukey’s test at p ≤ 0.05.

Chlorophylls (a, b and a + b) were also analysed in inner and outer lettuce leaves. As shown in Fig 4, the content of chlorophylls was altered by the effect of temperature (ambient or elevated temperature). CO_2_ levels (400 and 1000 ppm) and nutrient solution. In this sense, the chlorophyll a content in outer leaves varied from 1.95 (mg 100g^-1^ FW) (at elevated temperature, ambient CO_2_ and T-I nutrient solution) to 4.31 (mg 100g^-1^ FW) (at ambient temperature, elevated CO_2_ and T-IV nutrient solution), and in inner leaves, it varied between 4.30 (mg 100g^-1^ FW) (at elevated temperature, ambient CO_2_ and T-I nutrient solution) and 4.41 (mg 100g^-1^ FW) (at ambient temperature, elevated CO_2_ and T-IV nutrient solution). Chlorophyll b in the outer leaves ranged from 0.84 (mg 100g^-1^ FW) (at elevated temperature, ambient CO_2_ and T-I nutrient solution), to 4.75 (mg 100g^-1^ FW) (at ambient temperature, elevated CO_2_ and T-IV nutrient solution), and in inner leaves, from 2.90 (mg 100g^-1^ FW) (at elevated temperature, ambient CO_2_ and T-I nutrient solution) to 6.4 (mg 100g^-1^ FW) (at ambient temperature, elevated CO_2_ and T-IV nutrient solution). The total chlorophyll content in the outer leaves oscillated between 2.8 (mg 100g^-1^ FW) (at elevated temperature, ambient CO_2_ and T-I nutrient solution) and 9.06 (mg 100g^-1^ FW) (at ambient temperature, elevated CO_2_ and T-IV nutrient solution), and in inner leaves from 7.2 (mg 100g^-1^ FW) (at elevated temperature, ambient CO_2_ and T-I nutrient solution) to 10.81 (mg 100g^-1^ FW) (at ambient temperature, elevated CO_2_ and T-IV nutrient solution) (Fig 5). Table 2 shows that the maximal photochemical efficiency of PSII (Fv/Fm) followed the same pattern as the chlorophyll content. In this sense, Fv/ Fm varied from 0.62 in leaves from plants grown at ambient CO_2_ and fed with the T-I nutrient solution, and after being subjected to short-term heat stress, to 0.82 in conditions of ambient temperature, elevated CO_2_ and T-IV nutrient solution.

**Table 2:** Effect of heat stress, levels of CO_2_ and different NO3-/NH4+ ratios on photosystem II maximum efficiency (Fv/Fm) in outer leaves of baby-leaf lettuce cv ‘Derbi’.

Temperature	NO_3_^-^/ NH_4_^+^ treatment		Fv/Fm
Ambient temperature	T I	A CO_2_	0.76 ± 0.060^cd^
		E CO_2_	0.79 ± 0.037^abc^
	T II	A CO_2_	0.77 ± 0.033^cd^
		E CO_2_	0.79 ± 0.035^abc^
	T III	A CO_2_	0.79 ± 0.015^abc^
		E CO_2_	0.81 ± 0.011^ab^
	T IV	A CO_2_	0.80 ± 0.013^abc^
		E CO_2_	0.82 ± 0.004^a^
High temperature	T I	A CO_2_	0.62 ± 0.064^f^
		E CO_2_	0.67 ± 0.045^e^
	T II	A CO_2_	0.69 ± 0.044^e^
		E CO_2_	0.73 ± 0.012^d^
	T III	A CO_2_	0.77 ± 0.017^cd^
		E CO_2_	0.76 ± 0.007^cd^
	T IV	A CO_2_	0.79 ± 0.008^abc^
		E CO_2_	0.77 ± 0.003^bcd^

Different small letters represent significantly different mean values among the different temperatures and CO_2_ treatments and NO_3_^-^/NH_4_^+^ ratios according to Tukey’s test at p ≤ 0.05.

Short-term heat stress conditions resulted in an important decrease in chlorophyll a, chlorophyll b, and total chlorophyll concentration and Fv/Fm, by 50%, 57%, 53%, and 21%, respectively, as compared to that of the control (Figs 4 and 5, and Table 2). As with lipid peroxidation, these results also indicated that the cultivar studied was sensitive to heat stress. Similar results to ours have been observed by other authors in studies carried out in more than 41 cultivars of wheat (*Triticum aestivum* L.), cucumber (*Cucumis sativus*) seedlings, basil, Rice (*Oryza sativa* L.), Pea (*Pisum sativum* L.), Faba bean (*Vicia faba* L.) and Italian Lettuce (*Lactuca sativa* L. var. ramosa Hort) [26, 35, 50]. According to the literature, when plants were exposed to high temperatures, their photosystem II (PSII) photochemistry was affected. This was possibly due to a high production of ROS during temperature-stress conditions [8, 51]. Other studies support the hypothesis that a lower absorption of Mg gives rise to a greater activity of chlorophyllase, damaging the chloroplast apparatus and increasing the generation of ROS [52]. Additionally, in one study with lettuce, it was observed that plants stressed by high temperatures showed a lower absorption of Mg [53]. But it is worth mentioning that the decreased chlorophyll content in stressed plants may also be due to a deterioration in the biosynthesis of 5-aminolevulinic acid [8].

Fig 4 shows that after short-term heat stress, the decrease in chlorophyll b was more pronounced. This decrease was expected, and therefore, it is assumed that chlorophylls were degraded in different ways. In this sense, this remarkable decrease may have been caused by the fact that chlorophyll b was first converted to chlorophyll a. That conversion could have been performed by chlorophyll b reductase and 7-hydroxymethyl chlorophyll a reductase [54]. Both the elevated [CO_2_] and the nutrient solution alleviated the detrimental effects of heat stress produced on chlorophyll content and Fv/Fm in baby-leaf lettuce (Figs 4 and 5, Table 2). In studies carried out on tomato plants and coffee crops, it was found that the application of a high [CO_2_] attenuated the production of ROS species, favouring the mitigation of PSII photoinhibition in plants subjected to high temperatures [13, 55].

Within the plants under short-term heat stress, those treated with NO_3_^-^/ NH_4_^+^ at a 50/ 50 ratio exhibited the highest chlorophyll content and Fv/ Fm, showing a strengthening effect on the mitigation effects of short-term heat stress produced by the application of an elevated [CO_2_] (Figs 4 and 5, Tables 1 and 2). Early investigations have established that moderate proportions of NH_4_^+^ increased the content of chlorophylls. However, of note is the fact that the contribution of a high proportion of NH_4_^+^ in the nutrient solution led to a reduction of chlorophylls through chlorosis [56]. Thus, the relationship between NO_3_^-^/ NH_4_^+^ ratio in the nutrient solution and chlorophyll contents is also defined by a quadratic relationship [57].

Figs 4 and 5, Tables 1 and 2 indicate that the inner leaves contained a higher chlorophyll content and Fv/Fm than the outer leaves. It is proven that there is an association between stress-induced senescence and chloroplast stability, and chlorophyll metabolism [36, 58]. In this sense, abiotic stress induces premature leaf senescence as a result of the degradation of chlorophyll and other chloroplast components, causing a reduction in photosynthetic activity in plant leaves [59]. Our results did not reveal a specific mechanism, but they showed that senescence was delayed in heat-stressed plants, by the application of an elevated [CO_2_] and 50/ 50 NO_3_^-^/ NH_4_^+^ ratio.

### Total soluble protein content

**Fig 6:**
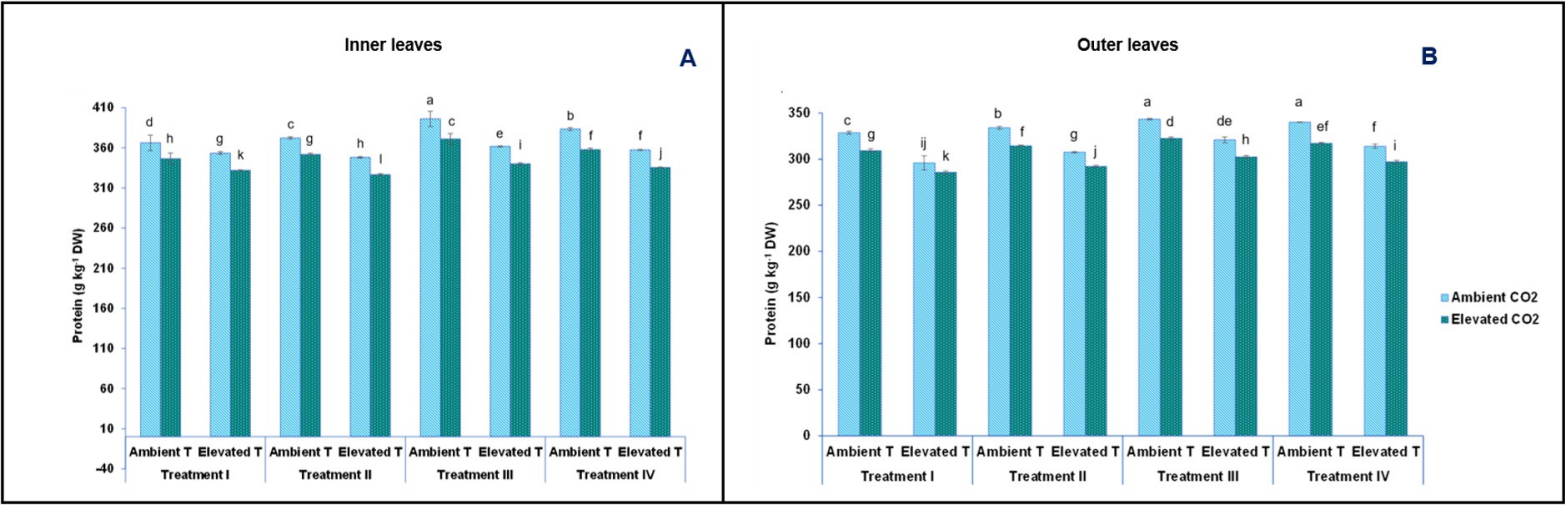
Effect of short-term heat stress on protein content (g kg^-1^ DW) in inner (A) and outer (B) leaves of the baby-leaf lettuce cv Derbi at two different levels of CO_2_ and at four different NO_3_^-^/ NH_4_^+^ treatments. Different letters are significantly different according to Tukey’s test at p ≤ 0.05.

As before, total soluble proteins were also measured in outer and in inner leaves from baby-leaf lettuce cv Derbi. Total soluble proteins varied significantly, with values ranging from 285.73 g kg^-1^ DW in the outer leaves at elevated temperatures, elevated [CO_2_], and T-I nutrient solution, to 395.88 g kg^-1^ DW in the inner leaves at ambient temperature, ambient [CO_2_] and the T-III nutrient solution (Fig 6).

As shown in Fig 6, the total soluble protein content in baby-leaf lettuce leaves decreased by 5% and 10% in outer and inner leaves, respectively, in response to heat stress, in those leaves grown under ambient CO_2_ and fed with control nutrient solution (T-I). These results were very similar to those previously reported for Italian Lettuce [60]. A substantial number of articles have stated that the exposure of plants to high temperatures above their threshold temperature (29.7°C) for their structural integrity and enzyme activity, can induce some irreversible damages to plant cells. Within this cell damage, we find the disruption of protein synthesis and activity, inactivation of some enzymes, and damage to membranes, all of which accelerate the rate of leaf senescence [61]. These cell damages generated are crucial mechanisms for the development of tolerance in plants. There are several fundamental signs of such mechanisms. One of them is the stimulation of the lipid signalling pathway and the other is the increase in cytoplasmic Ca^2+^, associated with the acquisition of tolerance through cytoskeletal reorganization [42]. These signals could be correlated with the inhibition of the enzymes rubisco and rubisco activase, which further damages the Calvin-Benson cycle (carbon fixation cycle in plants). As a result of a less carbon fixation, an excess of ROS species is obtained that inhibits any repair process that may occur in plants [62].

It was expected for the protein content to increase as [CO_2_] increased [63]. However, we obtained the opposite result. In fact, a reduction of 5% in the inner leaves and of 6% in the outer leaves was observed in plants when the only differentiating factor was the CO_2_ treatment. Therefore, these were plants grown under ambient temperature and with the T-I of nutrient treatment. This reduction could possibly be due to the N accumulating at that [CO_2_] (Fig 6), as it is known that N plays an important role in the biosynthesis of structural and enzymatic proteins [64]. However, this is not the only existing theory. Some studies ascribe this decrease in protein to a dilution effect resulting from a considerable increase in non-structural carbohydrates. In other manuscripts from this research team, it was observed that elevated [CO_2_] generated a large production of non-structural carbohydrates [53]. Moreover, other studies attribute such a decrease in total protein content to a decrease in rubisco. This lower concentration of rubisco is attributed not only to a reduction in transpiration and stomatal conductance, but also to a lower expression of photosynthesis-related genes, which are dependent on carbohydrates [65].

The nutrient treatments used in this study could be considered as stimulants of not only the synthesis of pigments, but also proteins. Since a higher content of NH_4_^+^ in the nutrient solution was supplied, a higher content of soluble proteins in baby-leaf lettuce cv Derbi was obtained (Fig 6 and Table 1). In this sense, for both outer and inner leaves, the highest content of soluble protein was obtained in plants grown under ambient temperature and CO_2_, and fed with the T-III nutrient solution (containing the 80/20 NO_3_^-^ / NH_4_^+^ ratio). These results are in agreement with those that claimed that supplying both forms of nitrogen in the 75/25 and 50/50 NO_3_^-^/ NH_4_^+^ ratio also led to the optimum total soluble proteins content in studies carried out with species such as lettuce [66], spinach [67], pepper [20], Chinese kale, and Chinese cabbage [31, 68]. Zhang et al. [20] reported that the highest protein synthesis obtained with the 75/25 NO_3_^-^/ NH_4_^+^ ratio was surely linked to a higher expression observed in glutamine synthetase (GS), glutamate synthases (GOGAT), and enzymatic activities, in nitrogen metabolism.

As with the chlorophyll content, when inner and outer leaves from plants grown and fed with the same conditions (ambient temperature and CO_2_, and fed with T-I nutrient solution) were compared, a lower (10%) content of total soluble proteins was obtained in the outer leaves than in the inner ones (Fig 6). Ougham *et al*. [69] informed that senescence was not only associated with a significant reduction in photosynthetic pigments, but also in protein content. This reinforces the conclusion from above: although our lettuce plants showed a premature senescence after short-term heat stress, an elevated [CO_2_] and a moderate content of NH_4_^+^ in the nutrient solution delayed that senescence.

## Conclusion

In summary, in the present work, we found that short-term heat stress drastically decreased the biomass, photosynthetic parameters (net photosynthetic rate, (A_CO2_) the intercellular/external CO_2_ concentration ratio (Ci/Ca) stomatal conductance (g_s_) and evapotranspiration (E)), photosystem II maximum efficiency (Fv/Fm), chlorophylls and proteins, and increased the lipid peroxidation in baby-leaf lettuce cv Derbi. This indicates that this baby-leaf lettuce is sensitive to heat stress. However, an elevated [CO_2_] alleviated the negative effects on photosynthetic parameters and photosynthetic pigments, proteins and lipid peroxidation produced in plants due to ROS accumulation. The positive effects of CO_2_ on plants was more pronounced when the plants were previously subjected to heat stress. This positive effect against heat stress on dry biomass, photosynthetic parameters, chlorophylls and lipid peroxidation was significantly strengthened by the application of T-IV nutrient solutions (containing 50/50 NO_3_^-^/ NH_4_^+^ ratio). Regarding the content of total soluble proteins, it was observed that the T-III treatment (80/20 NO_3_^-^/ NH_4_^+^ ratio) showed a greater heat stress mitigation. Thus, indeed, plant tolerance to short-term heat stress was considerably improved by the interaction between CO_2_ and the 80/20 or 50/50 NO_3_^-^ / NH_4_^+^ ratio in the nutrient solution.

This work sheds some light on the response of cv Derbi lettuce to a possible new strategy that could be useful for both dealing with the ever more frequent heat waves, and reducing nitrate contamination in the soil.
